# Aerosol Particle Size Influences the Infectious Dose and Disease Severity in a Golden Syrian Hamster Model of Inhalational COVID-19

**DOI:** 10.1089/jamp.2022.0072

**Published:** 2023-10-16

**Authors:** Jeremy A. Boydston, Jennifer Biryukov, John J. Yeager, Heather A. Zimmerman, Gregory Williams, Brian Green, Amy L. Reese, Katie Beck, Jordan K. Bohannon, David Miller, Denise Freeburger, Amanda Graham, Victoria Wahl, Michael C. Hevey, Paul A. Dabisch

**Affiliations:** National Biodefense Analysis and Countermeasures Center (NBACC), Operated by Battelle National Biodefense Institute for the US Department of Homeland Security, Frederick, Maryland, USA.

**Keywords:** COVID-19, dose response, infectious dose, particle size, SARS-CoV-2

## Abstract

**Background:**

Significant evidence suggests that SARS-CoV-2 can be transmitted via respiratory aerosols, which are known to vary as a function of respiratory activity. Most animal models examine disease presentation following inhalation of small-particle aerosols similar to those generated during quiet breathing or speaking. However, despite evidence that particle size can influence dose-infectivity relationships and disease presentation for other microorganisms, no studies have examined the infectivity of SARS-CoV-2 contained in larger particle aerosols similar to those produced during coughing, singing, or talking. Therefore, the aim of the present study was to assess the influence of aerodynamic diameter on the infectivity and virulence of aerosols containing SARS-CoV-2 in a hamster model of inhalational COVID-19.

**Methods:**

Dose–response relationships were assessed for two different aerosol particle size distributions, with mass median aerodynamic diameters (MMADs) of 1.3 and 5.2 μm in groups of Syrian hamsters exposed to aerosols containing SARS-CoV-2.

**Results:**

Disease was characterized by viral shedding in oropharyngeal swabs, increased respiratory rate, decreased activity, and decreased weight gain. Aerosol particle size significantly influenced the median doses to induce seroconversion and viral shedding, with both increasing ∼30-fold when the MMAD was increased. In addition, disease presentation was dose-dependent, with seroconversion and viral shedding occurring at lower doses than symptomatic disease characterized by increased respiratory rate and decreased activity.

**Conclusions:**

These results suggest that aerosol particle size may be an important factor influencing the risk of COVID-19 transmission and needs to be considered when developing animal models of disease. This result agrees with numerous previous studies with other microorganisms and animal species, suggesting that it would be generally translatable across different species. However, it should be noted that the absolute magnitude of the observed shifts in the median doses obtained with the specific particle sizes utilized herein may not be directly applicable to other species.

## Introduction

Substantial evidence suggests that SARS-CoV-2 can be transmitted via respiratory aerosols. Both infectious virus^1–3^ and viral RNA^4–7^ have been detected in air samples collected in the vicinity of COVID-19 patients. Similarly, virus has been detected in the exhaled breath collected directly from COVID-19 patients.^8,9^ The size distribution of droplets emitted by an infected individual is known to vary for different respiratory activities.^10–12^ Relatively low concentrations of smaller particle sizes are generated during quiet breathing, whereas higher concentrations of larger particles are generated during speaking or coughing.^13^ Particle size affects the site of deposition in the respiratory tract, with smaller particles having a higher probability of reaching the alveolar regions, while larger particles are more likely to deposit in the upper respiratory tract.^14–16^

Previous studies have demonstrated that particle size influences not only the regional deposition within the respiratory tract, but also the amount of pathogen required to cause infection and disease presentation, with the amount of pathogen required to cause disease increasing as particle size increases.^17–22^ Thus, the potential for aerosol transmission of disease may be influenced by the size distribution of respiratory particles generated during various activities.

Many studies, utilizing a range of different routes of exposure, have examined disease presentation in animal models of COVID-19. Several studies have examined disease presentation following inhalation of small-particle aerosols (*d*_a_ between 1 and 3 μm) in nonhuman primates^23–26^ and hamsters.^27^ However, no studies were identified that examined the infectivity of larger particle aerosols, similar to those produced during coughing, singing, or talking. Therefore, the aim of the present study was to assess the influence of aerodynamic diameter on the infectivity and virulence of aerosols containing SARS-CoV-2 in a hamster model of inhalational COVID-19.

## Materials and Methods

### Ethics statement

All research was conducted in compliance with the Animal Welfare Act and other federal statutes and regulations relating to animals and experiments involving animals and adheres to the principles stated in the Guide for the Care and Use of Laboratory Animals and approved by both the National Biodefense Analysis and Countermeasures Center (NBACC) Institutional Animal Care and Use Committee and, when applicable, the Department of Homeland Security (DHS) Compliance Assurance Program Office. The facility where this research was conducted is fully accredited by AAALAC International and maintains a Public Health Service Humane Care and Use of Laboratory Animals (Policy) assurance.

### Virus and viral assays

SARS-CoV-2 isolate hCoV-19/USA/CA_CDC_5574/2020 (VOC-202012/01; B.1.1.7 lineage; Alpha variant) was obtained from the Centers for Disease Control and Prevention. This virus was received as passage 2 and was propagated once in Vero cells to yield passage 3 working stocks that were used in the present study. SARS-CoV-2 was propagated in and microtitrated on Vero (ATCC CCL-81) cells. Vero cells were cultured in complete growth media at 37°C and 5% CO_2_, as previously described.^28^ Infected Vero cell monolayers and culture supernatants were freeze-thawed, clarified, and prepared as described previously.^29^ Virus was aliquoted and stored at −80°C before use in experiments. SARS-CoV-2/USA/CA_CDC_5574/2020 (p3) was sequenced and found to match the consensus sequence for SAMN18527802. All work with SARS-CoV-2 was performed in biosafety level 3 containment.

The concentration of infectious virus in each stock and in aerosol samples was estimated using a Vero cell-based microtitration assay as described in detail previously^24^ and reported as median tissue culture infectious doses (TCID_50_) per mL. Microtitration assay plates were read between 4 and 7 days postinfection.

### Animal exposures and postexposure monitoring

Male and female golden Syrian hamsters (HsdHan:AURA), 3–5 weeks of age, and certified by the vendor (Envigo) to be free of specific rodent pathogens, were housed singly in individually ventilated cages (GR900; Tecniplast). All hamsters were confirmed to be seronegative to SARS-CoV-2 by ELISA before study initiation (EI 2606-9601 G; Euroimmun, Inc.).

**FIG. 1.**
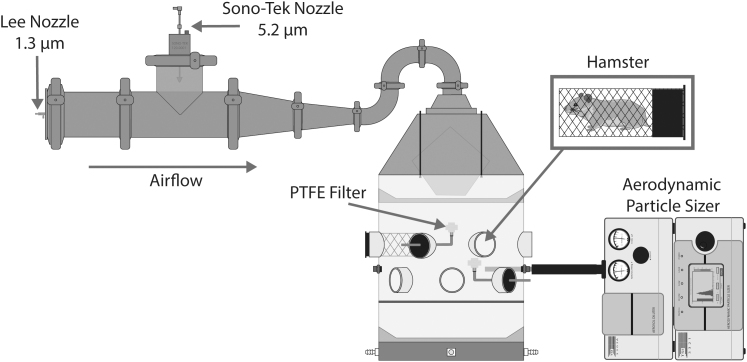
Whole-body hamster exposure system. Aerosols were generated with an air assist nozzle for small particle aerosols (MMAD: 1.3 μm) or an ultrasonic nozzle for larger particle aerosols (MMAD: 5.2 μm). After equilibration, SARS-CoV-2-containing aerosols were drawn into a whole-body exposure chamber where hamsters were individually housed. Aerosols were sampled during the exposure to determine concentration of infectious virus and the particle size distribution of the generated aerosols using PTFE filters and an APS, respectively. APS, aerodynamic particle sizer; MMAD, mass median aerodynamic diameters; PTFE, polytetrafluoroethylene.

For both small- and large-particle exposures, seven groups of eight golden Syrian hamsters were exposed to varying concentrations of aerosols containing SARS-CoV-2 to estimate the median doses for different endpoints. Exposure of hamsters was performed using a custom whole-body rodent exposure chamber described previously,^30^ with hamsters individually housed in mesh whole-body exposure tubes (Fig. 1).

An air assist nozzle (PN: IAZA5200415K; The Lee Company), supplied with 16 L/min of dry compressed air and 50 μL/min of viral suspension, was used for generating small-particle aerosols. The equilibrated mass median aerodynamic diameter (MMAD) measured in the breathing zone using an Aerodynamic Particle Sizer (APS, Model 3321; TSI, Inc.) equipped with a diluter (1:100, Model 3302A; TSI, Inc.) was 1.34 ± 0.04 μm with a geometric standard deviation (GSD) of 1.55 ± 0.02. For large-particle aerosols, a 120 kHz ultrasonic aerosol generator (Sono-Tek Corp.) supplied with 150 μL/min of viral suspension was utilized. The equilibrated MMAD measured in the breathing zone using an APS was 5.19 ± 0.28 μm with a GSD of 1.28 ± 0.01. These particle size distributions are expected to result in significant differences in the respiratory deposition pattern in hamsters, with the small-particle aerosols expected to result in significant deposition in the pulmonary region, whereas the large-particle aerosols would be expected to deposit a majority of inhaled aerosols in the conducting airways and upper respiratory tract.^31–33^

Aerosols were generated into clean air flowing through the plenum at 21 L/min. Following equilibration in the plenum, generated aerosol was delivered to the exposure chamber. The equilibrium relative humidity in the exposure system during aerosol generation was 35%–40%. Exposure duration was 10 minutes. Aerosol concentration in the breathing zone was estimated using 25 mm polytetrafluoroethylene filters (22-3708; SKC, Inc.), which have been shown previously to efficiently sample airborne SARS-CoV-2.^34^

Following exposure, filters were removed from the system, placed into 50 mL conical tubes containing 5 mL of gMEM, and vortexed to resuspend collected virus. The resuspended samples were assayed for infectivity using a viral microtitration assay. The aerosol concentration of infectious virus was estimated as the product of the viral titer, in TCID_50_/mL, and the resuspension volume, in mL, divided by the total volume of air sampled. The aerosol spray factors, calculated as the ratio of the measured aerosol concentration to the concentration of the viral suspension used for aerosol generation, for small- and large-particle aerosols, were 1.6 × 10^−6^ ± 9.1 × 10^−7^ and 1.9 × 10^−6^ ± 3.8 × 10^−7^, respectively.

The inhaled dose of infectious virus for each animal, in TCID_50_, was estimated as the product of the infectious aerosol concentration and the total volume of air inhaled during the exposure period. The total volume of air inhaled during the exposure period was estimated as the product of the respiratory minute volume and the exposure duration. An estimate of the respiratory minute volume of 200 mL/min was utilized based on preliminary testing with hamsters and a whole-body, double-chambered rodent plethysmograph (EMKA SCIREQ, Canada) and IOX version 2.10 software (EMKA SCIREQ).

Hamsters were monitored for development of disease for 28 days postexposure. Temperature was measured twice daily from day −3 through day 14 postexposure, and then once daily through day 28 postexposure, using implanted subcutaneous transponder chips (IPTT-300; Bio Medic Data Systems, Inc.). Respiratory rate was measured by cage side observation each morning through day 14 postexposure before other activities to ensure that the hamsters were not in an excited state due to handling/disturbance by animal care technicians. Bodyweights were recorded from day −3 through day 28 postexposure.

Hamsters had access to running wheels continuously during the pre- and postexposure periods for measurement of activity. Activity was measured continuously from day −3 to day 14 postexposure using an activity wheel (Kaytee Silent Spinner Wheel, 6.5″; Kaytee Products, Inc.) paired with a rotational counter and magnetic proximity sensor (CH7N and S3391; Fargo Controls, Inc.) mounted to the exterior of each cage. Total daily rotations were manually recorded. Instances where the wheels were obstructed by nesting material were noted to ensure that artificial decreases in activity were not measured.

Oropharyngeal swabs were collected pre-exposure and on days 2, 4, 6, 8, 10, 14, 21, and 28 postexposure for analysis of viral shedding. Swabs were analyzed for infectious virus using a viral microtitration assay and for viral RNA using a reverse transcription quantitative real-time PCR assay (see Supplementary Data for more details).

On day 28 postexposure, a blood sample was collected, after which animals were euthanized in accordance with the AVMA Guidelines on Euthanasia.^35^ Seroconversion was assessed using the Euroimmun SARS-CoV-2 S1 ELISA kit (El 2606-9601 G; Euroimmun, Inc.), with the substitution of goat anti-Syrian hamster IgG H&L horseradish peroxidase conjugate at 0.05 μg/mL (Catalog No: ab6892; Abcam) as the secondary antibody. Absorbance was read on a SpectraMax iD5 Microplate Reader. Samples were considered positive if the optical density of terminal samples was greater than three standard deviations above the prechallenge sample.

### Data analysis

Dose–infectivity relationships were estimated using a generalized linear model with a probit link function using log_10_ transformed inhaled doses (JMP v. 16.0.0; SAS Institute, Inc.). Time-course analyses of running wheel activity, respiratory rate, and bodyweight were performed using one- or two-way ANOVA, as appropriate (GraphPad Prism v. 9.0.0; GraphPad Software LLC.).

## Results

**FIG. 2.**
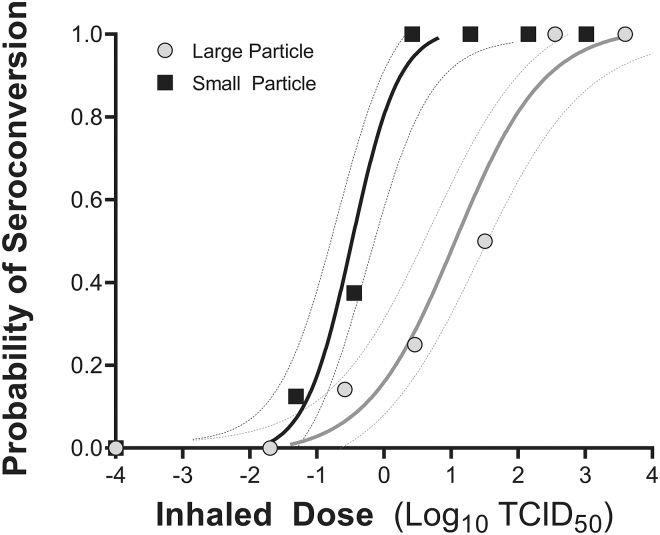
Dose–response relationships for hamsters exposed to aerosols containing SARS-CoV-2 with MMADs of 1.3 and 5.2 μm. The dose–response curve for seroconversion for hamsters exposed to small and large particle aerosols containing SARS-CoV-2 Alpha variant, along with 85% confidence intervals for each, is shown. Symbols indicate the fractional responses for each dose group from Table 1. TCID_50_, tissue culture infectious doses.

Seroconversion was dose-dependent for groups exposed to small- and large-particle aerosols containing the alpha variant of SARS-CoV-2 (Tables 1, 2 and Fig. 2). Particle size was a significant factor influencing the dose–response relationships (*p* < 0.0001 by likelihood ratio test [LRT]), and the 85% confidence intervals (CIs) for the median doses for seroconversion, which have been shown to be a reliable indicator of significance for independent dose–response models, do not overlap (Table 2 and Fig. 2). The slopes of the dose–response relationships for the two different particle sizes were similar (*p* = 0.12 by LRT).

**Table 1.** Summary of Inhaled Doses and Seroconversion

Small particle (MMAD: 1.3 μm)			Large particle (MMAD: 5.2 μm)		
Inhaled dose (TCID_50_)	Inhaled dose (log_10_ TCID_50_)	Seroconversion	Inhaled dose (TCID_50_)	Inhaled dose (log_10_ TCID_50_)	Seroconversion
1038.39	3.0	8/8	3943.66	3.6	8/8
141.47	2.2	8/8	354.49	2.5	8/8
19.27	1.3	8/8	31.86	1.5	4/8
2.63	0.4	8/8	2.86	0.6	2/8
0.39	−0.4	3/8	0.26	−0.6	1/7^a^
0.05	−1.3	1/8	0.02	−1.6	0/8
0	0	0/8	0	Media	0/8

^a^ Group size was seven due to removal of one hamster before challenge.

MMAD, mass median aerodynamic diameter; TCID_50_, tissue culture infectious doses.

**Table 2.** Median Dose for Seroconversion for Hamsters Exposed to Aerosolized SARS-CoV-2

Particle size	D_50, seroconversion_	
	TCID_50_	log_10_ TCID_50_
Small (MMAD 1.3 μm)	**0.3** 85% CI: −0.2 to 1.5 95% CI: 0.12 to 1.0	**−0.48** 85% CI: −0.76 to 0.17 95% CI: −0.91 to 0.00
Large (MMAD 5.2 μm)	**11.5** 85% CI: 4.3 to 31.6 95% CI: 2.8 to 50.1	**1.06** 85% CI: 0.63 to 1.50 95% CI: 0.44 to 1.70

Boldface represents ID_50_ values. CI, confidence interval.

All animals that seroconverted also had detectable infectious virus in oral swabs at some point during the postexposure period, and there were no animals that shed virus but did not seroconvert. Therefore, a dose–response analysis for shedding, where an animal was considered positive if infectious virus was detected in any post-exposure sample, results in identical median doses to those for seroconversion for both the particle size groups.

**FIG. 3.**
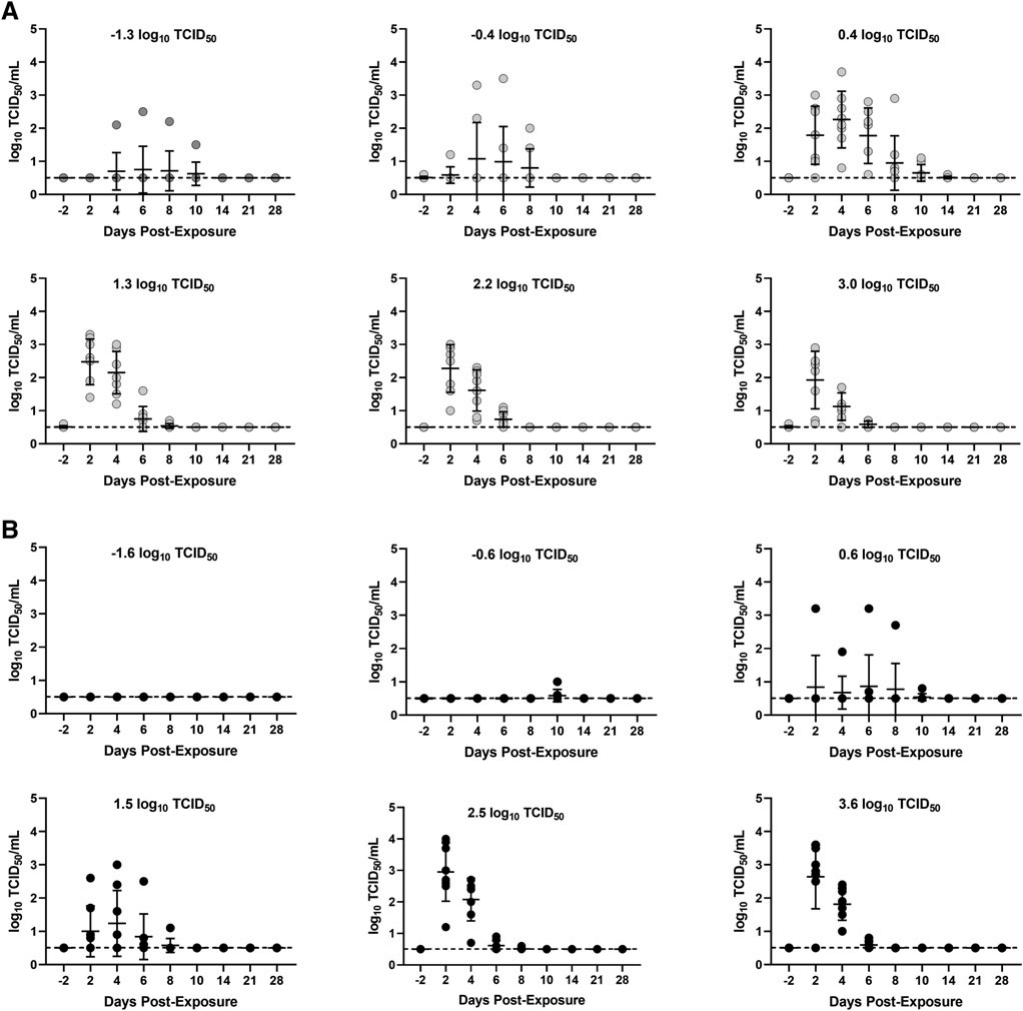
Shedding of infectious virus in oropharyngeal swabs. **(A)** Shedding of infectious virus in oropharyngeal swabs from hamsters exposed to different inhaled doses of small particle aerosols containing SARS-CoV-2 Alpha variant. **(B)** Shedding of infectious virus in oropharyngeal swabs from hamsters exposed to different inhaled doses of large particle aerosols containing SARS-CoV-2 Alpha variant. Individual points represent each hamster, and line and error bars represent mean and SD, respectively. Dashed line represents limit of quantification. Grey and black circles represent individual data points/values.

**FIG. 4.**
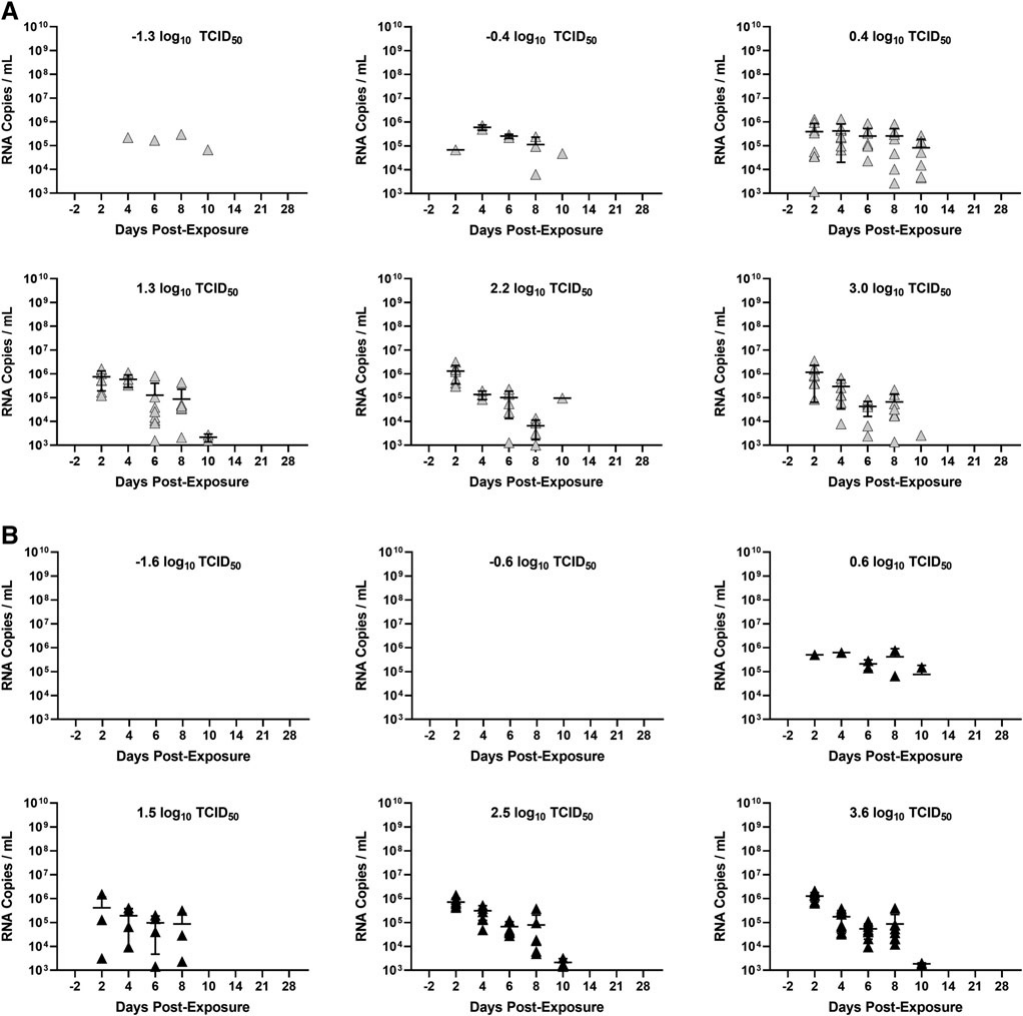
Shedding of viral RNA in oropharyngeal swabs. **(A)** Shedding of viral RNA in oropharyngeal swabs from hamsters exposed to different inhaled doses of small particle aerosols containing SARS-CoV-2 Alpha variant. **(B)** Shedding of viral RNA in oropharyngeal swabs from hamsters exposed to different inhaled doses of large particle aerosols containing SARS-CoV-2 Alpha variant. Individual points represent each hamster, and line and error bars represent mean and SD, respectively. Grey and black circles represent individual data points/values.

Higher levels of infectious virus and viral RNA were detected in oral swabs on days 2 and 4 followed by a drop on day 6 for most animals and undetectable levels by days 10 to 14 (Figs. 3 and 4). While the probability of shedding occurring postexposure was dose-dependent, there was not a strong correlation between the inhaled dose and the peak concentration of infectious virus detected in oral swabs (*r*^2^ < 0.11 for both the particle size groups; Supplementary Data). Across all dose groups, the median peak infectious titer in oral swabs from animals exposed to small-particle aerosols was 2.5 log_10_ TCID_50_/mL (interquartile range [IQR]: 1.9–3.7 log_10_ TCID_50_/mL), which was not significantly different than hamsters exposed to large-particle aerosols, which had a median peak infectious titer of 2.7 log_10_ TCID_50_/mL (IQR: 2.5–4.0 log_10_ TCID_50_/mL; *p* = 0.13 when compared by Mann–Whitney test).

Similarly, there was not a strong correlation between the inhaled dose and the number of days infectious virus was detected in oral swabs (*r*^2^ < 0.14 for both the particle size groups; Supplementary Data). The duration of shedding was also not significantly different between the small- and large-particle-exposed groups (*p* = 0.15 by Mann–Whitney test), with a median number of shedding days for both small- and large-particle-exposed hamsters of 3.0 days (IQR: 2–3 days for both groups).

Viral RNA in oropharyngeal swabs was detected over a similar period to that observed for infectious virus (Fig. 4). Similar peak levels of SARS-CoV-2 RNA were detected in small- and large-particle-exposed hamsters, with a median of 5.9 log_10_ RNA copies/mL (IQR: 5.7–6.2 log_10_ RNA copies/mL) for small-particle-exposed animals and a median of 5.9 log_10_ RNA copies/mL (IQR: 5.7–6.1 log_10_ RNA copies/mL) for large-particle-exposed animals (*p* = 0.86 by Mann–Whitney test). The duration of shedding of viral RNA was also similar for small- and large-particle-exposed hamsters, with a median of 4.0 days (IQR: 3.2–4.0 days) for small-particle-exposed animals and 4.0 days (IQR: 4.0–5.0 days) for large-particle-exposed animals (*p* = 0.18 by Mann–Whitney test).

Across all dose and particle size groups, the duration of shedding of viral RNA (median: 4.0 days, IQR: 4.0–4.3 days) was slightly but significantly longer than that of infectious virus (median 3.0 days, IQR: 2.0–3.0 days; *p* < 0.0001 by Mann–Whitney test).

Baseline running wheel activity for the three pre-exposure days was normally distributed and averaged 27,985 ± 6,229 revolutions per day across all hamsters. Decreases in activity, defined as a greater than a three standard deviation decrease from baseline, were observed for the five higher dose groups of small-particle-exposed hamsters (−0.4 to 3.0 log_10_ TCID_50_), but only the two highest dose groups for large-particle-exposed hamsters (2.5 to 3.6 log_10_ TCID_50_) (Supplementary Data). The probability of a decrease in running wheel activity in animals exposed to small-particle aerosols was dose dependent, with a median inhaled dose for a decrease in activity in small-particle-exposed animals of 1.8 log_10_ TCID_50_ (85% CI: 1.3–2.4 log_10_ TCID_50_; 95% CI: 1.1–2.7 log_10_ TCID_50_), which is significantly greater than those estimated for seroconversion and viral shedding when compared by an overlap of 85% CIs (Supplementary Data).

While the response in the large-particle-exposed groups also appears to have dose dependence, no dose group had greater than 3/8 animals with significant decreases in activity, complicating estimation and comparison of dose–response relationships for the different particle size groups. Therefore, dose–response comparisons as a function of size are not presented for decreases in activity.

**FIG. 5.**
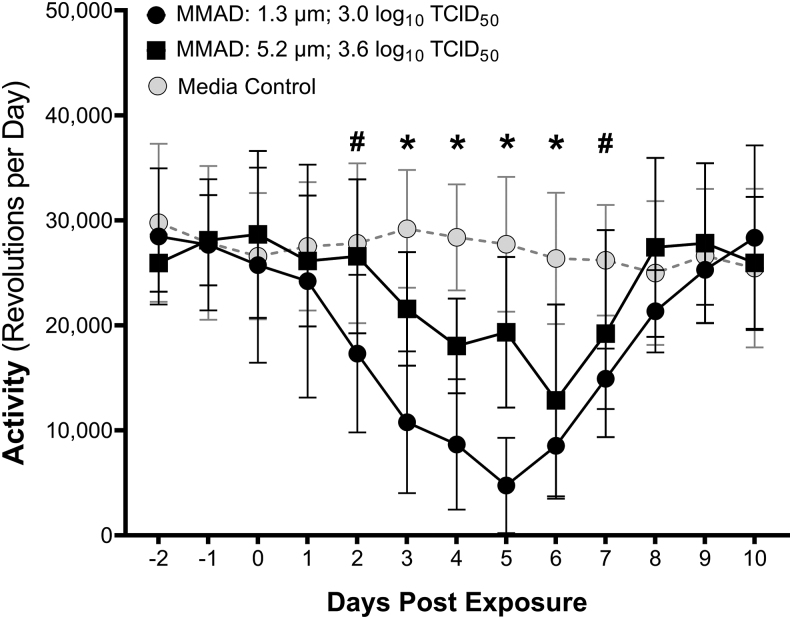
Running wheel activity for hamsters exposed to aerosols containing SARS-CoV-2 with MMADs of 1.3 and 5.2 μm. Groups of hamsters exposed to small (MMAD: 1.3 μm; black circles) or large (MMAD: 5.2 μm; black squares) particle aerosols containing SARS-CoV-2 displayed reduced running wheel activity postexposure relative to media-exposed controls (gray circles). Two-way ANOVA of the effects of exposure type (i.e., large particle SARS-CoV-2, small particle SARS-CoV-2, or media) and time postexposure on activity suggests that both factors influenced activity (*p* < 0.002). Animals exposed to both large and small particle aerosols containing SARS-CoV-2 had significant decreases in activity relative to media exposed on days 3–6 postexposure (denoted by *). Animals in the small particle-exposed group also had decreased activity on days 2 and 7 postexposure (denoted by #). Activity levels returned to those of media-exposed controls on day 8 postexposure. The decrease in activity for small particle-exposed animals was greater than that observed for large particle-exposed animals on days 3–5 postexposure (*p* < 0.012).

A comparison of running wheel activity over time for animals receiving similar inhaled doses of large- and small-particle aerosols, as well as control animals exposed to aerosolized culture media, is shown in Figure 5. Two-way ANOVA of the effects of exposure type (i.e., large particle SARS-CoV-2, small particle SARS-CoV-2, or media) and time postexposure suggests that both factors influenced running wheel activity (*p* < 0.002). SARS-CoV-2-exposed animals had significant decreases in activity relative to media-exposed controls beginning on day 2 postexposure (*p* < 0.038). Activity levels returned to those of media-exposed controls on day 8 postexposure. The decrease in activity for small-particle-exposed animals was greater than that observed for large-particle-exposed animals on days 3–5 postexposure (*p* < 0.012).

Across all groups, the baseline respiration rate for hamsters was 79 ± 12 breaths per minute. Increases in respiratory rate, defined as a greater than a three standard deviation increase from baseline, were observed for the four higher dose groups of small-particle-exposed hamsters (0.4 to 3.0 log_10_ TCID_50_) and the two highest dose groups exposed to large-particle aerosols (2.5 to 3.6 log_10_ TCID_50_; Supplementary Data). The probability of an increase in respiratory rate was dose dependent in animals exposed to both small- and large-particle aerosols. The median inhaled dose for an increase in respiratory rate was 1.2 log_10_ TCID_50_ (85% CI: 0.8–1.5 log_10_ TCID_50_; 95% CI: 0.7–1.7 log_10_ TCID_50_) in small-particle-exposed animals and 1.7 log_10_ TCID_50_ (85% CI: 1.3–2.1 log_10_ TCID_50_; 95% CI: 1.1–2.4 log_10_ TCID_50_) in large-particle-exposed animals.

**FIG. 6.**
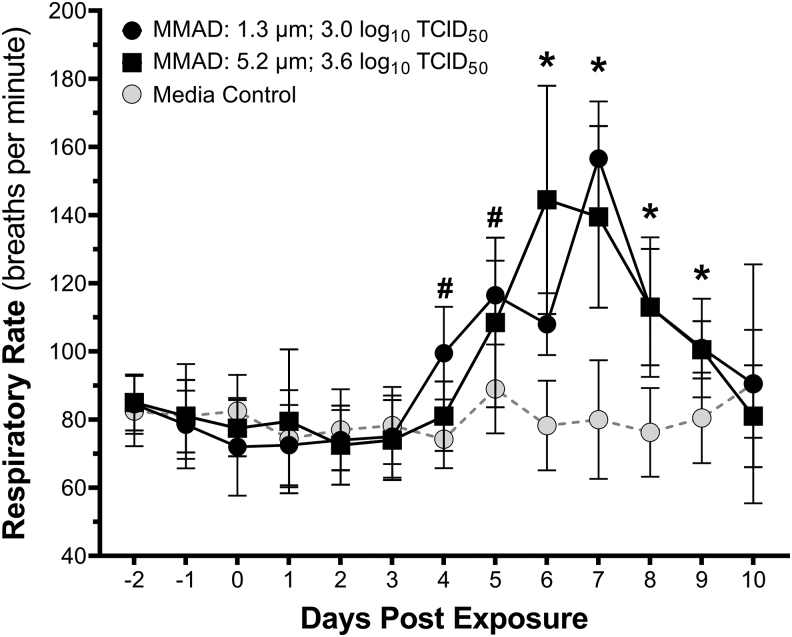
Respiratory rate for hamsters exposed to aerosols containing SARS-CoV-2 with MMADs of 1.3 and 5.2 μm. Groups of hamsters exposed to small (MMAD: 1.3 μm; black circles) or large (MMAD: 5.2 μm; black squares) particle aerosols containing SARS-CoV-2 displayed an increased respiratory rate postexposure relative to media-exposed controls (gray circles). Two-way ANOVA of the effects of exposure type (i.e., large particle SARS-CoV-2, small particle SARS-CoV-2, or media) and time postexposure on activity suggests that both factors influenced respiratory rate (*p* < 0.0001). Animals exposed to both large and small particle aerosols containing SARS-CoV-2 had significant increases in respiratory rate relative to media-exposed controls on days 6–9 postexposure (denoted by *). The respiratory rate of the small particle-exposed group was also greater than the media-exposed controls on day 4 and 5 postexposure (denoted by #). Respiratory rates returned to those of media-exposed controls on day 10 postexposure. Respiratory rate also differed between the small and large particle-exposed groups on days 4 and 6 postexposure (*p* < 0.05).

Particle size did not significantly influence the dose–response relationships (*p* = 0.25 by LRT), and the 85% CIs for the median doses for the two size groups overlapped. As with decreases in activity, these median estimates are significantly greater than those estimated for seroconversion and viral shedding when compared by an overlap of 85% CIs (Supplementary Data). A comparison of respiratory rate over time for animals receiving similar inhaled doses of large- and small-particle aerosols, as well as control animals exposed to aerosolized culture media, is shown in Figure 6.

**FIG. 7.**
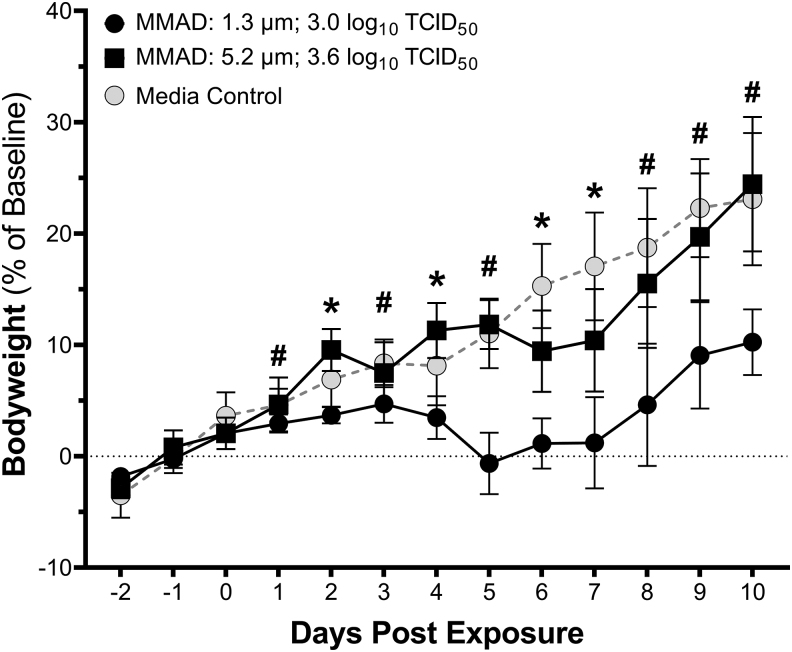
Body weights for hamsters exposed to aerosols containing SARS-CoV-2 with MMADs of 1.3 and 5.2 μm. Groups of hamsters exposed to small (MMAD: 1.3 μm; black circles) or large (MMAD: 5.2 μm; black squares) particle aerosols containing SARS-CoV-2 had decreased weight gain postexposure relative to media-exposed controls (gray circles). Two-way ANOVA of the effects of exposure type (i.e., large particle SARS-CoV-2, small particle SARS-CoV-2, or media) and time postexposure on activity suggests that both factors influenced respiratory rate (*p* < 0.0001). Weight gain in animals exposed to both large and small particle aerosols containing SARS-CoV-2 was significantly less than media-exposed controls on days 2, 4, 6, and 7 postexposure (denoted by *). Weight gain in the small particle-exposed group was also decreased relative to the media-exposed controls on days 1, 3, 5, 8, 9, and 10 postexposure (denoted by #). Weight gain also differed between the small and large particle-exposed groups on days 2 and 4–10 postexposure (*p* < 0.05).

Body weight data are presented in Figure 7, expressed as percent of the day 0 weight. Weight gain was significantly reduced in small-particle-exposed animals relative to media-exposed controls over the entire postexposure period (*p* < 0.005; two-way ANOVA by exposure type and day postexposure), and relative to large-particle-exposed animals on days 2 and 4–10 postexposure (*p* < 0.005). Weight loss was also observed in some of the small-particle-exposed animals from days 5 to 9 postexposure. Weight gain was also significantly reduced in large-particle-exposed animals relative to media-exposed controls on days 2, 4, 6, and 7 postexposure (*p* < 0.05). Weight loss was not observed in any animals in the large-particle- or media-exposed groups.

Hamsters were subcutaneously implanted with a transponder chip to record daily body temperature. No significant changes in body temperature were observed throughout the study for either small- or large-particle aerosol groups.

## Discussion

The present study demonstrates that aerosol particle size is an important factor influencing dose–response relationships and disease presentation in a hamster model of inhalational COVID-19. The median inhaled dose needed for seroconversion and induction of viral shedding increased ∼30-fold when the MMAD of inhaled aerosols was increased from 1.3 to 5.2 μm. These data suggest that aerosol particle size may be an important factor influencing the risk of disease transmission for COVID-19, and needs to be considered when developing animal models of disease. Given that this increase in particle size would be expected to shift regional deposition of inhaled particles from the lower to upper respiratory tract,^31–33^ these results also suggest that the site of aerosol deposition within the respiratory tract may be an important determinant of disease presentation.

This result is in agreement with numerous previous studies with other microorganisms and animal species, suggesting that it would be expected to be generally translatable across different species. However, it should be noted that the absolute magnitude of the observed shifts in median doses obtained with the specific particle sizes utilized with the hamster model in the present study may not be directly applicable to other species, as regional deposition patterns in the respiratory tract are a function of particle size, host size, and respiratory parameters.

Numerous studies with other microorganisms have demonstrated differences in disease presentation and the dose needed to cause disease as a function of particle size and regional deposition pattern within the respiratory tract.^17–20,22^ The increase in the median doses for seroconversion and viral shedding observed in the present study as MMAD increased is consistent with the results of these previous studies with other microorganisms and reinforces the importance of aerosol particle size and route of exposure as a consideration in the development of animal models of infectious disease. It should be noted that doses in the present study are reported as inhaled doses and do not account for the deposition fraction of inhaled material within the respiratory tract.

Previous studies in hamsters have demonstrated that both the total and regional deposition fractions would be expected to differ for aerosols with the MMADs utilized in the present study. Specifically, Raabe et al. reported that the total deposition fraction for hydrophobic monodisperse aerosols of aluminosilicate was ∼20% for aerosols with an MMAD of 1 μm and 100% for aerosols with an MMAD of 5.3 μm.^32^ Thus, in the present study, the relative difference between the total doses received by the small- and large-particle-exposed groups would increase if deposition fraction was accounted for, resulting in an even greater difference in the estimated median doses for seroconversion and shedding as a function of size than already reported in the present study.

The study by Raabe et al. also reported that the regional deposition patterns differed for aerosols with MMADs of 1 and 5.3 μm, with greater pulmonary deposition for aerosols with a 1 μm MMAD and greater deposition in the upper respiratory and gastrointestinal tracts for aerosols with a 5 μm MMAD. However, it should be noted that the composition of the inhaled aerosols in the present study, which utilized hygroscopic viral culture medium, differed significantly from Raabe et al., which utilized hydrophobic aluminosilicate, and the degree to which this difference affects the total and regional deposition fractions is unclear.^31,32^

While the mechanisms responsible for the observed particle size-dependent differences remain unclear, it is possible that a difference in regional deposition patterns of inhaled particles plays a role given the physiological differences between the different regions of the respiratory tract. Particles that deposit in the upper respiratory tract are cleared by mucociliary clearance, unlike particles that deposit in more distal regions of the lung where macrophages are involved in phagolysosomal destruction, and it is possible that differences in the efficiency of these clearance mechanisms may play a role. Differences in tissue tropism could also play a role, although SARS-CoV-2 gains entry via interaction with ACE-2, which is expressed in both the upper and lower respiratory tracts and numerous other tissues, suggesting a broad cellular tropism.^36,37^

In the present study, inhalational disease in hamsters exposed to either small- or large-particle aerosols was characterized by dose-dependent viral shedding in oropharyngeal swabs, increased respiratory rate, decreased activity, and decreased weight gain. Disease presentation was influenced by dose, with seroconversion and shedding occurring at lower doses than symptomatic disease characterized by an increased respiratory rate and a decreased running wheel activity.

Quantitative activity measurement via running wheels is a sensitive metric for assessing disease severity in rodent models.^38–40^ Gerhards et al. demonstrated a marked decrease in activity in hamsters following intranasal inoculation with dose of 4.5 log_10_ TCID_50_ of SARS-CoV-2.^41^ Similarly, in the present study, decreases in running wheel activity were observed in animals exposed to both large- and small-particle aerosols containing SARS-CoV-2. The probability of a decrease in running wheel activity in animals exposed to small-particle aerosols was dose dependent. However, while the response in the large-particle-exposed groups also appeared to be dose dependent, no dose group had greater than 3/8 animals with decreases in activity, which complicated estimation of the dose–response relationship as well as comparison with the small-particle-exposed group.

Differences in the magnitude of the response were observed between the small- and large-particle groups receiving similar inhaled doses, suggesting that the magnitude of the decrease in activity may be particle size and/or dose dependent. These data also suggest that measurement of activity in hamsters exposed to SARS-CoV-2 provides a sensitive and quantitative indicator of disease severity.

Weight loss has been reported in hamster models of COVID-19 exposed by the intranasal route.^42,43^ While decreased weight gain was observed consistently in the present study, weight loss was only observed in a few animals exposed to small-particle aerosols. Weight loss appears to be dose dependent in previous studies utilizing the intranasal route of exposure,^43,44^ suggesting that the lack of consistent weight loss in the present study may be due to the lower doses utilized in the present study relative to those in previous studies utilizing intranasal exposure. The average weight gain in small-particle-exposed animals was significantly slower than that observed in large-particle-exposed animals to a similar dose, again suggesting that particle size and/or dose may influence the magnitude of the response and disease severity.

Increased respiratory rate, or tachypnea, has been reported in human cases of COVID-19.^45^ In the present study, increases in respiratory rate were observed 4 to 9 days postexposure in both the small- and large-particle-exposed groups, and the probability of an increase in respiratory rate postexposure was dose dependent in both groups. However, unlike other metrics of disease presentation, the response did not appear to be particle size dependent, as the median doses and magnitude of the increase were similar between the two particle size groups.

Finally, a previous study reported temperature fluctuations following intranasal inoculation of 10^5^ TCID_50_ of SARS-CoV-2, specifically a drop in temperature on days 3 and 4 postexposure.^46^ However, in the present study, no changes in body temperature were observed in any of the groups exposed to aerosols containing SARS-CoV-2. The absence of temperature fluctuations in the present study could be due to the dose as the maximum inhaled dose utilized in the present the study was ∼100-fold less than the amount of virus needed to cause a decrease in body temperature in the previous study. In addition, temperature measurements were only recorded twice daily, and it is possible that the low frequency of these measurements precluded detection of short periods of temperature fluctuation.

## Supplementary Material

Supplemental data

## Disclaimer

The views and conclusions contained in this document are those of the authors and should not be interpreted as necessarily representing the official policies, either expressed or implied, of DHS or the U.S. Government. The DHS does not endorse any products or commercial services mentioned in this presentation. In no event shall the DHS, BNBI, or NBACC have any responsibility or liability for any use, misuse, inability to use, or reliance upon the information contained herein. In addition, no warranty of fitness for a particular purpose, merchantability, accuracy, or adequacy is provided regarding the contents of this document.

## Notes

Reviewed by:

Gehard Scheuch

Phillip Kuehl
