# Supplementary material for: Aerosol Particle Size Influences the Infectious Dose and Disease Severity in a Golden Syrian Hamster Model of Inhalational COVID-19
Source: J Aerosol Med Pulm Drug Deliv. 2023 Oct 16;36(5):235–45. doi: 10.1089/jamp.2022.0072 (PMC10615081; doi:10.1089/jamp.2022.0072)
Supplement: Supplemental data [file Suppl_Data.docx]

Aerosol particle size influences the infectious dose and disease severity in a golden Syrian hamster model of inhalational COVID-19

Jeremy A. Boydston*, Jennifer Biryukov, John J. Yeager, Heather A. Zimmerman, Gregory Williams, Brian Green, Amy L. Reese, Katie Beck, Jordan K. Bohannon, David Miller, Denise Freeburger, Amanda Graham, Victoria Wahl, Michael C. Hevey, and Paul A. Dabisch

National Biodefense Analysis and Countermeasures Center (NBACC), Operated by Battelle National Biodefense Institute for the US Department of Homeland Security, Frederick, Maryland, USA

*Corresponding author: jeremy.boydston@nbacc.dhs.gov

Notice: This manuscript has been authored by Battelle National Biodefense Institute, LLC under Contract No. HSHQDC-15-C-00064 with the U.S. Department of Homeland Security. The United States Government retains and the publisher, by accepting the article for publication, acknowledges that the United States Government retains a non-exclusive, paid up, irrevocable, world-wide license to publish or reproduce the published form of this manuscript, or allow others to do so, for United States Government purposes.

**SUPPLEMENTARY MATERIALS.**

**RT-qPCR.** The target of the PCR assay is a conserved region of the viral RNA-dependent RNA polymerase (RdRp) gene. SARS-CoV-2 RNA was quantified using a one-step RT-PCR MasterMix with Platinum Taq DNA polymerase and an Applied Biosystems 7500 Fast real time PCR instrument. Briefly, viral RNA was isolated and purified from test samples using the Qiagen Viral RNA Mini kit following the manufacturer instructions. Reactions were set up by combining 15 µL of master mix with 5 µL of viral RNA. PCR master mix was composed of sterile, molecular-grade water, 1x SuperScript reaction mix, 1x SuperScript reverse transcriptase, 0.2 µM forward and reverse primers, and 0.1 µM 6-carboxyflorescein (FAM)-labeled fluorescent probe. The primers and probe sequences were based on those previously published by Corman et al. (46) but were modified to replace the redundant bases with consensus bases. The primer and probe sequences are as follows: forward primer (5’ - GTG AAA TGG TCA TGT GTG GCG G – 3’), reverse primer (5’ – CAA ATG TAA AAA ACA CTA TTA GCA TA – 3’), 6-carboxyfluorescein (FAM) labeled, double quencher probe (5’ - /56-FAM/ AGG TGG AAC /ZEN/ CTC ATC AGG AGA TGC C/31AbkFQ/ - 3’). Each reaction plate contained a standard curve, ranging from 10^1^ to 10^7^ RNA copies per 5 µL, based on a synthetic RNA positive control representing the assay’s target amplicon (BioSynthesis, Inc.). Cycling conditions were run as follows: hold for 50 °C for 30 min, 95 °C for 10 min, and then 40 cycles of 95 °C for 15 s and 60 °C for 1 min. Quantification was determined by the number of cycles required to cross a threshold of 0.02 (values reported as threshold cycles [Ct]). Viral RNA copies/mL of sample were interpolated from the standard curve for each plate from each sample with a Ct ≤ 30.

**Table S1. Summary of Response Data for Small Particle Exposed Groups**

| **Inhaled Dose** (TCID_50_) | **Inhaled Dose** (log_10_ TCID_50_) | **Seroconversion** | **Shedding in Oral Swabs** | **Decreased Activity** | **Increased Respiratory Rate** |
| --- | --- | --- | --- | --- | --- |
| 1038.39 | 3.0 | 8/8 | 8/8 | 7/8 | 8/8 |
| 141.47 | 2.2 | 8/8 | 8/8 | 5/8 | 7/8 |
| 19.27 | 1.3 | 8/8 | 8/8 | 3/8 | 4/8 |
| 2.63 | 0.4 | 8/8 | 8/8 | 0/8 | 2/8 |
| 0.39 | -0.4 | 3/8 | 3/8 | 0/8 | 0/8 |
| 0.05 | -1.3 | 1/8 | 1/8 | 1/8 | 0/8 |
| 0 | Media only | 0/8 | 0/8 | 0/8 | 0/8 |

**Table S2. Summary of Response Data for Large Particle Exposed Groups**

| **Inhaled Dose** (TCID_50_) | **Inhaled Dose** (log_10_ TCID_50_) | **Seroconversion** | **Shedding in Oral Swabs** | **Decreased Activity** | **Increased Respiratory Rate** |
| --- | --- | --- | --- | --- | --- |
| 3943.66 | 3.6 | 8/8 | 8/8 | 3/8 | 8/8 |
| 354.49 | 2.5 | 8/8 | 8/8 | 2/8 | 8/8 |
| 31.86 | 1.5 | 4/8 | 4/8 | 1/8 | 4/8 |
| 2.86 | 0.6 | 2/8 | 2/8 | 0/8 | 2/8 |
| 0.26 | -0.6 | 1/7* | 1/7* | 0/7* | 1/7* |
| 0.02 | -1.6 | 0/8 | 0/8 | 1/8 | 0/8 |
| 0 | Media only | 0/8 | 0/8 | 0/8 | 0/8 |

*Group size was seven due to removal of one hamster prior to challenge.


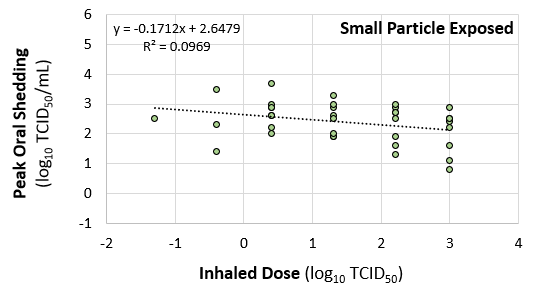


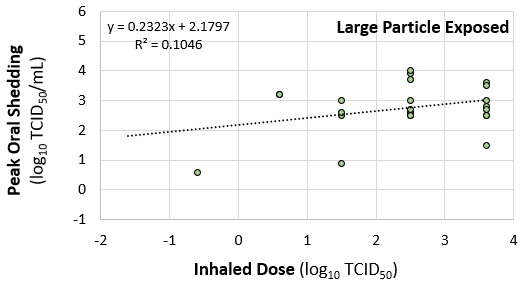


**Figure S1. Peak Shedding of Infectious Virus in Oral Swabs Versus Dose.**  (Top) Small particle exposed animals. (Bottom) Large particle exposed animals.


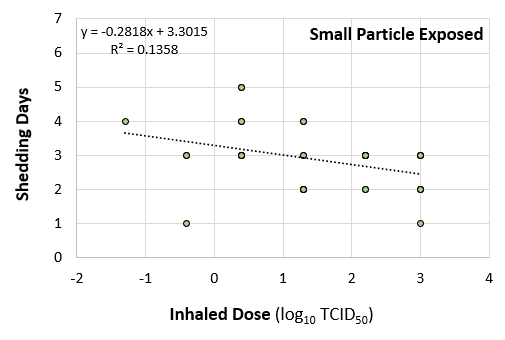


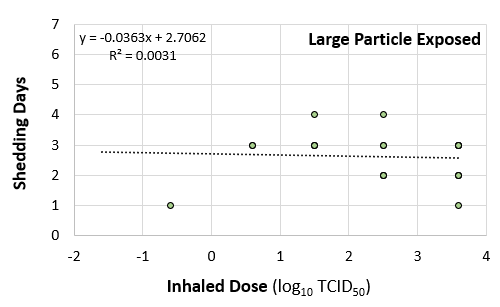


**Figure S2. Number of Shedding Days of Infectious Virus in Oral Swabs Versus Dose.**  (Top) Small particle exposed animals. (Bottom) Large particle exposed animals.

##
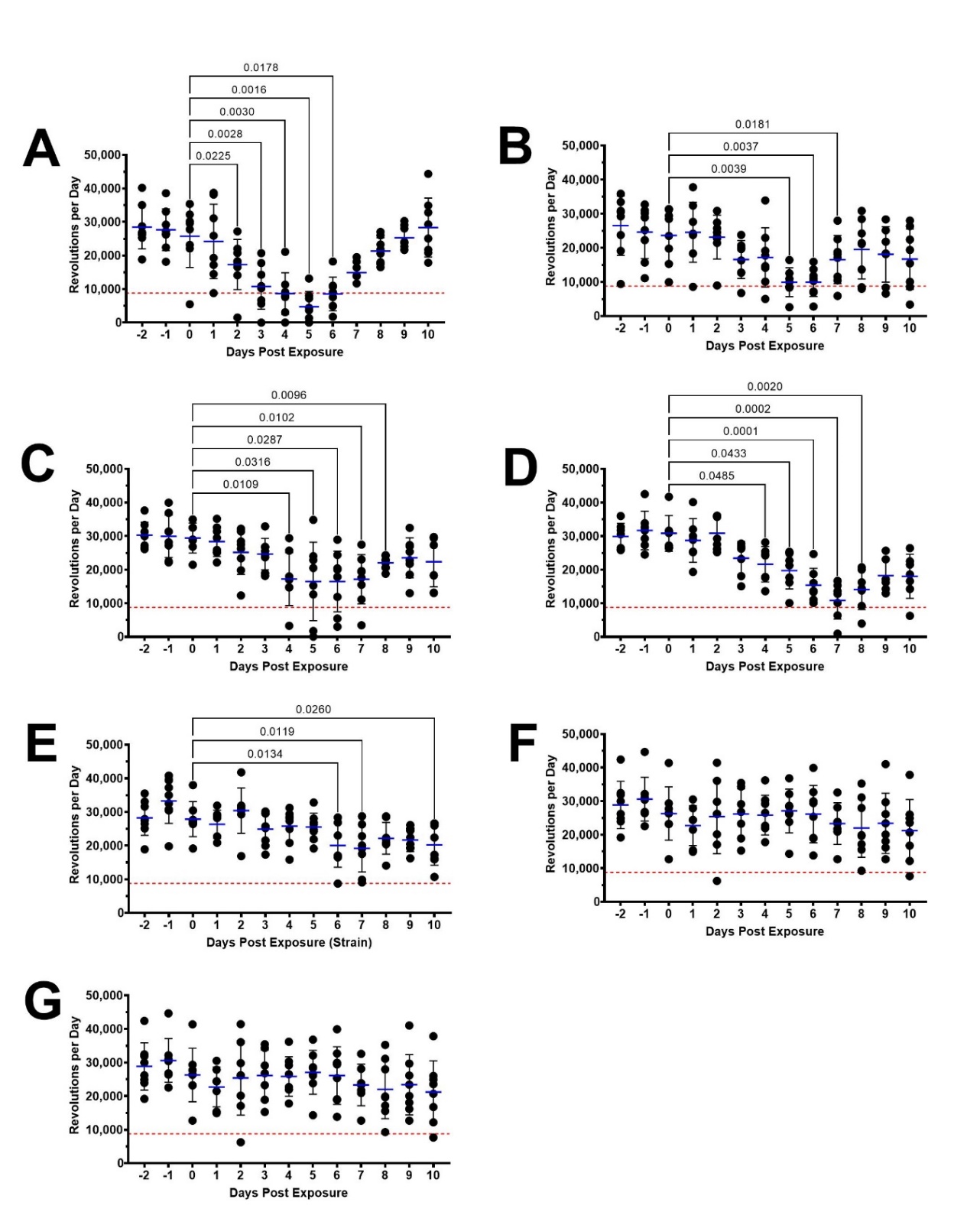


**Figure S3: Activity following challenge with small particle (1.3µm) SARS-CoV-2 Alpha.** A one-way ANVOA was used to compare day 0 to day 1 -10 postexposure, days with significant decrease are shown with P values (black lines). (A) 3.0 log TCID_50_ (B) 2.2 log TCID_50_ C) 1.3 log TCID_50_(D) 0.4 log TCID_50_ (E) -0.4 log TCID_50_ (F) -1.3 log TCID_50_ (G) Media Control. Blue line represents mean with SD, red dashed line represents 3 SD below the mean baseline.


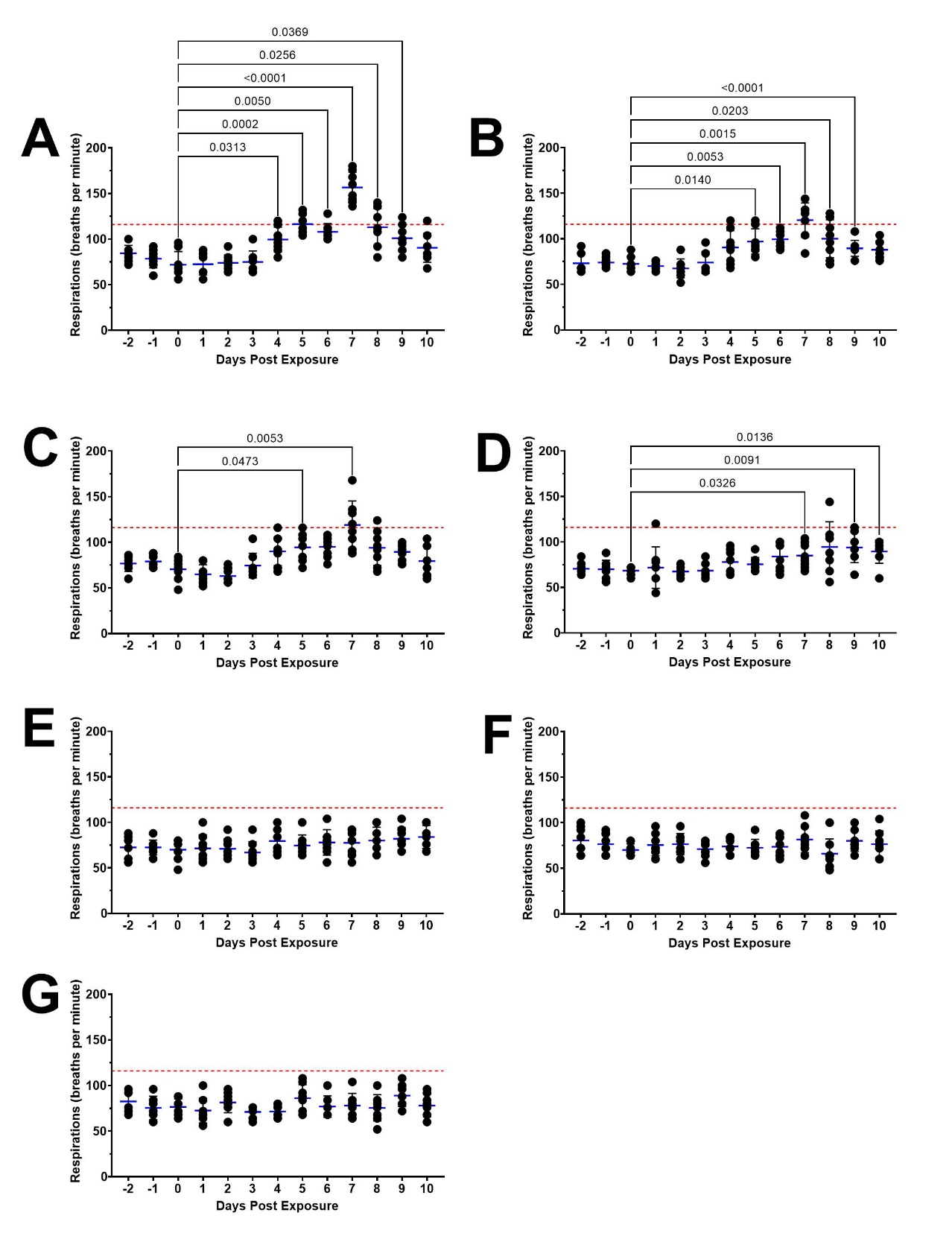


**Figure S4: Respirations following challenge with small particle (1.3µm) SARS-CoV-2 Alpha.** A one-way ANVOA was used to compare day 0 to day 1 -10 postexposure, days with significant increase are shown with P values (black lines). (A) 3.0 log TCID_50_ (B) 2.2 log TCID_50_ (C) 1.3 log TCID_50_(D) 0.4 log TCID_50_ (E) -0.4 log TCID_50_ (F) -1.3 log TCID_50_ (G) Media Control. Blue line represents mean with SD, red dashed line represents 3 SD above the mean baseline.


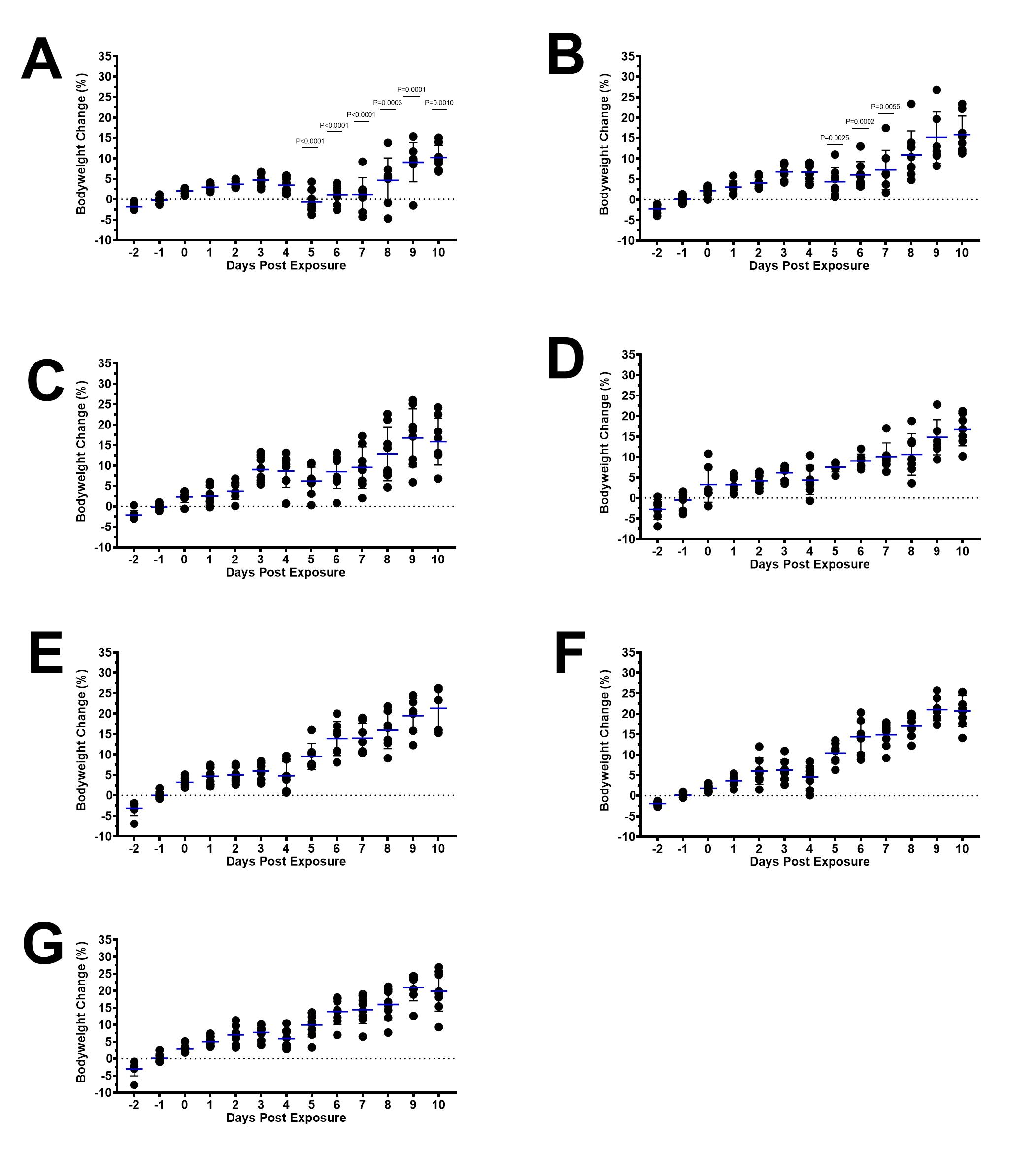


**Figure S5: Percent change in bodyweight following challenge with small particle (1.3µm) SARS-CoV-2 Alpha.** A one-way ANVOA was used to compare the media controls day matched with dose groups A, B, C, D, E, F for day 0 -10 postexposure, days with significant difference are shown with P values (black lines). (A) 3.0 log TCID_50_ (B) 2.2 log TCID_50_ (C) 1.3 log TCID_50_(D) 0.4 log TCID_50_ (E) -0.4 log TCID_50_ (F) -1.3 log TCID_50_ (G) Media Control. Blue line represents mean with SD, gray dashed line represents zero change.


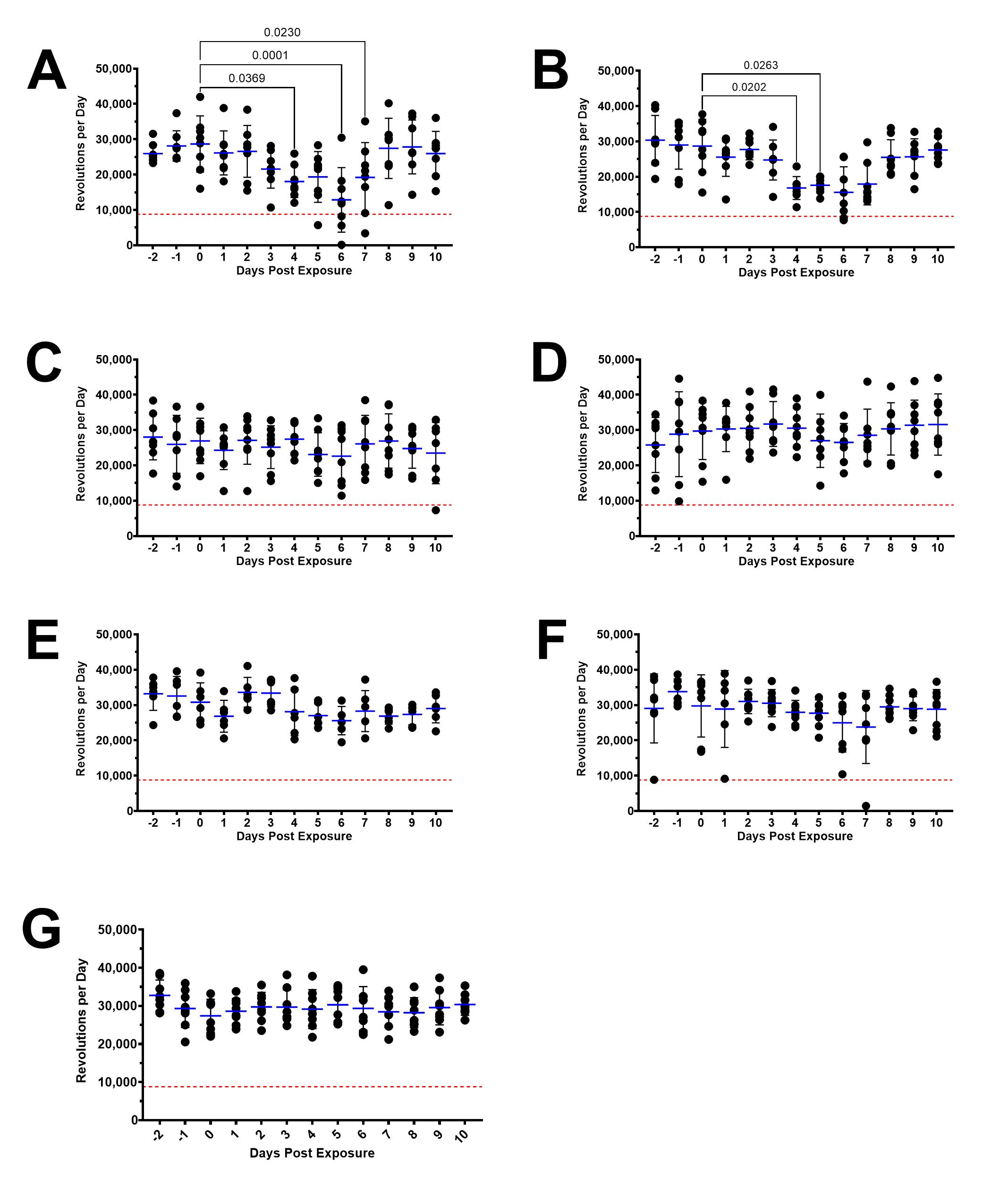


**Figure S6: Activity following challenge with large particle (5.2µm) SARS-CoV-2 Alpha.** A one-way ANVOA was used to compare day 0 to day 1 -10 postexposure, days with significant decrease are shown with P values (black lines). (A) 3.6 log TCID_50_ (B) 2.5 log TCID_50_ (C) 1.5 log TCID_50_(D) 0.5 log TCID_50_ (E) -0.6 log TCID_50_ (F) -1.6 log TCID_50_ (G) Media Control. Blue line represents mean with SD, red dashed line represents 3 SD below the mean baseline.


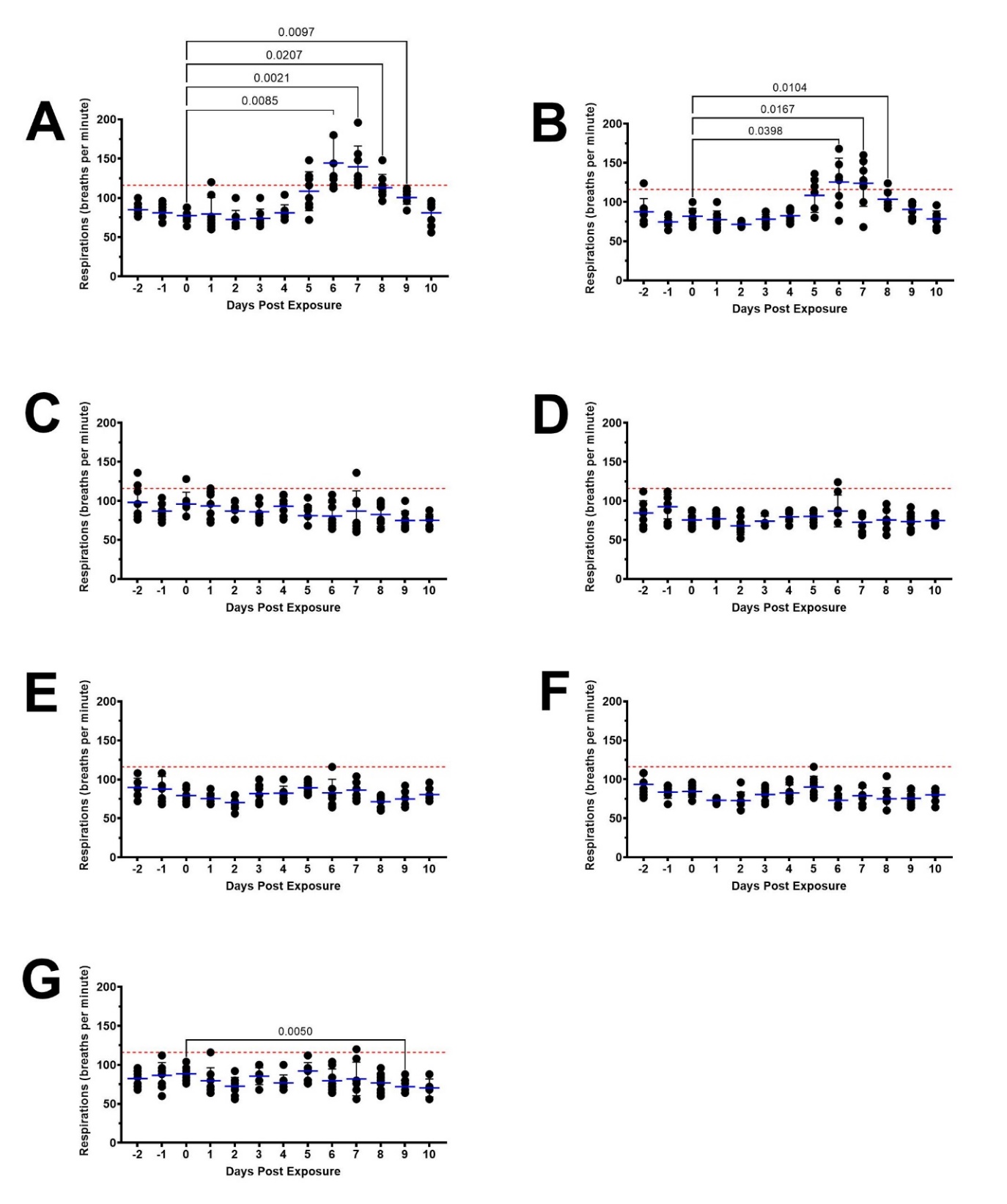


**Figure S7: Respiratory rate following challenge with large particle (5.2 µm) SARS-CoV-2 Alpha.** A one-way ANVOA was used to compare day 0 to day 1 -10 postexposure, days with significant increase are shown with P values (black lines). (A) 3.6 log TCID_50_ (B) 2.5 log TCID_50_ (C) 1.5 log TCID_50_(D) 0.5 log TCID_50_ (E) -0.6 log TCID_50_ (F) -1.6 log TCID_50_ (G) Media Control. Blue line represents mean with SD, red dashed line represents 3 SD above the mean baseline.


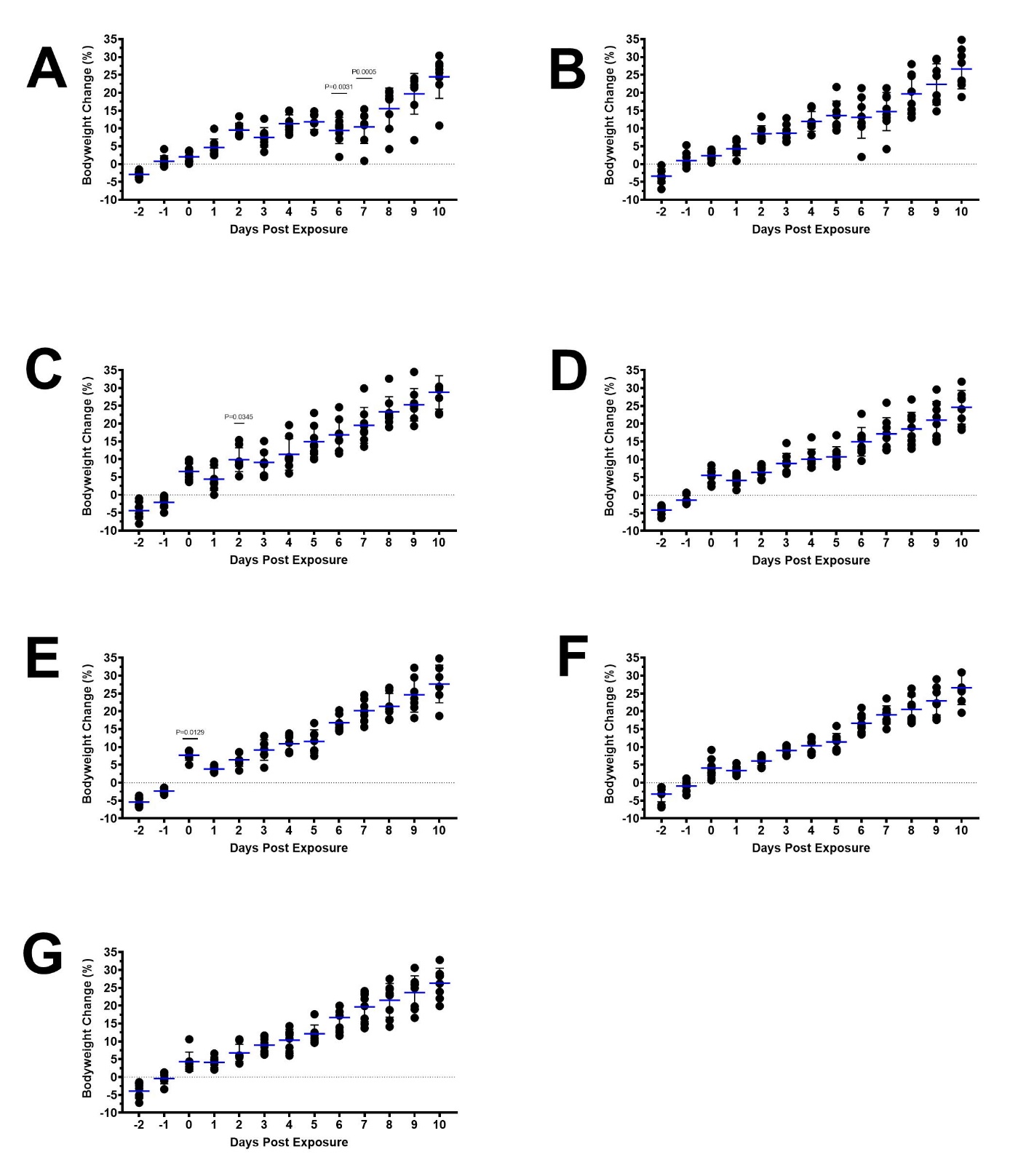


**Figure S8: Percent change in bodyweight following challenge with large particle (5.2µm) SARS-CoV-2 Alpha.** A one-way ANVOA was used to compare the media controls day matched with dose groups A, B, C, D, E, F for day 0 -10 postexposure, days with significant difference are shown with P values (black lines). (A) 3.6 log TCID_50_ (B) 2.5 log TCID_50_ (C) 1.5 log TCID_50_(D) 0.5 log TCID_50_ (E) -0.6 log TCID_50_ (F) -1.6 log TCID_50_ (G) Media Control. Blue line represents mean with SD, gray dashed line represents zero change.
